# Could combination of radical hysterectomy and radiation effective in the treatment of primary cervical signet ring cell carcinoma?: A rare case report

**DOI:** 10.1016/j.ijscr.2022.107083

**Published:** 2022-04-13

**Authors:** Gatot Purwoto, Kartiwa Hadi Nuryanto, Taufik Agung Wibowo, Tantri Hellyanti, Danny Maesadatu Syaharutsa, Kelli Julianti

**Affiliations:** a Department of Obstetrics and Gynecology, Faculty of Medicine University of Indonesia, Dr. Cipto Mangunkusumo Hospital, Jakarta, Indonesia; b Department of Radiology, Faculty of Medicine University of Indonesia, Dr. Cipto Mangunkusumo Hospital, Jakarta, Indonesia; c Department of Anatomical Pathology, Faculty of Medicine University of Indonesia, Dr. Cipto Mangunkusumo Hospital, Jakarta, Indonesia; d Department of Obstetrics and Gynecology, Faculty of Medicine University of Indonesia, Jakarta, Indonesia

**Keywords:** Signet-ring cell, Cervical carcinoma, Radical hysterectomy, Radiation

## Abstract

**Introduction:**

Primary signet-ring cell carcinoma (PSRCC) of the uterine cervix mostly occurs due to the metastasis from the primary organ, such as the gastrointestinal tract or breast. This case describes the cervical PSRCC and its management as a rare case.

**Presentation of case:**

The 39-year-old female came to undergo cancer screening. Visual Inspection with Acetic Acid (VIA) examination result was negative with the feature of severe cervicitis, and then the patient was given Trichloroacetic Acid (TCA) procedure. Three weeks after TCA's procedure, speculum examination found morphological features of cervix malignancy. Cervical biopsy examination showed mucinous adenocarcinoma, signet ring cell variant, with lymphovascular invasion. Endometrial microcurettage specimen do not contain tumor mass.

**Discussion:**

The risk factor for cervical cancer in this patient was early sexual intercourse. We suggested stage IB2 cervical cancer because the tumor size was 2 cm until 4 cm, there was no spreading to nearby lymph nodes nor distant sites. Then patient had performed a radical hysterectomy procedure with ovary transposition and a series of radiation therapy. The patient was in good condition and no metastases were found in the imaging study.

**Conclusion:**

The treatment of PSRCC of the uterine cervix is challenging. It was established from intraoperative findings, histopathology, and immunohistochemistry examination. The radical hysterectomy adjunct to radiation was effective in the treatment of the PSRCC of the uterine cervix.

## Highlights

• Primary Signet Ring Cell Carcinoma (PSRCC) of the uterine cervix is a rare case, where in general, Signet Ring Cell Carcinoma (SRCC) as a subtype of mucinous adenocarcinoma and it is originated from gastrointestinal tract malignancy
• PSRCC of uterine cervix was established from intraoperative finding and pathology examination
• Combination of radical hysterectomy and radiation is effective in the treatment of PSRCC of uterine cervix

## 1 Introduction

Signet ring cell carcinoma of the cervix is an extremely rare variant of cervical carcinoma. It most frequently represents a metastasis from primary gastric tumor and only rarely a primary cervical malignancy. Even it can infiltrate all pelvic abdominal organs, including the genitalia organ. The most common type of cervical carcinoma is squamous cell carcinoma. Adenocarcinoma accounts for 10–25% of cervical carcinomas; with the most frequent histological type is HPV-associated endocervical adenocarcinoma, usual type, followed by mucinous adenocarcinoma. Signet ring cell carcinoma is a rare variant of mucinous carcinoma [1]. Clinical findings of tumor in ovary or uterine was a secondary from the primary tumor in intestinal, that usually called Krukenberg tumor [2]. SRCC could be found in non-colorectal organs, which could be confirmed by pathology examination, which the mechanism is not much explained. Cervical carcinoma in stage I could be successfully treated with either radical hysterectomy or radiation with or without concurrent chemotherapy, with primary chemoradiation [3]. This case will present the primary tumor case in the cervix without any findings in another organ and according to pathology examination, SRCC was founded.

## 2 Case Illustration

**Fig. 1:**
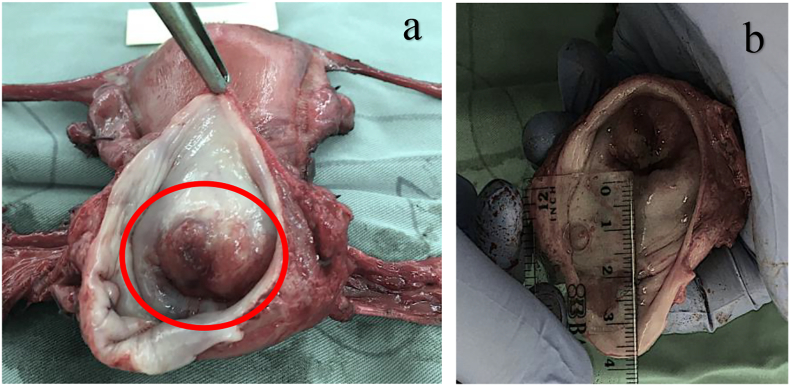
(a). Macroscopic appearance of the cervix and with exophytic mass as the primary tumor; (b) upper part of the vagina.

A 39-year-old woman complained about post-coital bleeding one year ago. The menstrual cycle was normal and sometimes, she had intermittent lower abdominal pain. She married when she was 16 years old, has only one sexual partner, and has two children. She has not been vaccinated for HPV. Visual Inspection with Acetic Acid (VIA) examination result was negative with the feature of severe cervicitis, and then the patient was given Trichloroacetic Acid (TCA) procedure. Three weeks after TCA's procedure, speculum examination found morphological features of cervix malignancy. Speculum examination revealed a large exophytic cervical tumor sized 3 × 3 × 2 cm with atypical blood vessels, fragile, easy bleeding. Biopsy examination found mucinous adenocarcinoma, signet ring cell variant, with lymphovascular invasion. Both whole abdomen Magnetic Resonance Imaging (MRI) with contrast and thorax x-ray were performed, and no abnormalities from those imaging studies (Fig. 1).

**Fig. 2:**
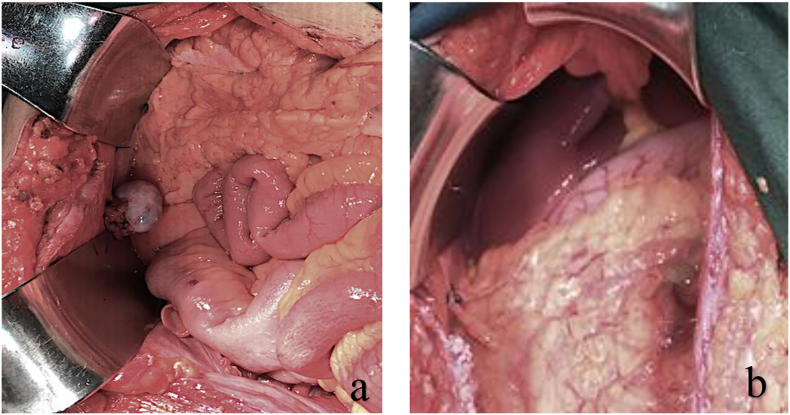
(a) and (b) Abdominal exploration during procedure

The patient was performed a radical hysterectomy, right salpingectomy, left salpingo-oophorectomy, transposition of right ovary, and bilateral pelvic lymphadenectomy. During the procedure, the exploration in the spleen, liver, colon, gaster, omentum, and diaphragma were discovered smooth without nodules (Fig. 2).

The specimens were sent for histopathology examination. Seven days after surgery patient was discharged. Histopathology examination revealed mucinous adenocarcinoma, signet ring cell variant of the cervix. There was lymphovascular invasion and metastasis in one of the 11 right pelvic lymph nodes. While no metastatic deposit found in the left pelvic lymph nodes. Microscopically, the tumor mass is mainly arranged in non-cohesive clusters with abundant intracytoplasmic mucin and peripherally located nuclei, which resemble a signet ring. In addition, glandular and solid patterns were also found.

Malignant tumor cells invade almost the full thickness of the cervix. There was no invasion of the vagina or bilateral parametrium. According to FIGO staging, the stage was 1B2. The patient received 25 fractions external radiation therapy and two fractions for internal radiation therapy.

The patient routinely visits the oncology gynecology clinic for a routine follow up. In six months and a year after this patient had completed a series of radiotherapy, she was reported in good condition and got a MRI study. The MRI study found no pathological signal in the tumor bed cervix and vaginal punctum. There was no lymphadenopathy in parailliaca and paraaortic lymph nodes (Fig. 4).

**Table:** 

Timeline	
Year	Description
2019	• August: VIA examination and TCA therapy was given • September: suggested to do repeated VIA examination in next six months • November: suspected cervical cancer
2020	• March: patient was performed radical hysterectomy, right salpingectomy, left salpingo-oophorectomy, transposition of right ovary, and bilateral pelvic lymphadenectomy • April to July: The patient received 25 fractions external radiation therapy and two fractions internal radiation therapy • October: MRI with contrast taken after series of radiotherapy was completed

## 3 Discussion

The risk factors for cervical cancer were sexually transmitted infection (STI) and reproductive and sexual factors. STI comprised infection of HPV type 16 and 18. Moreover, it could be exacerbated by Human Immunodeficiency Virus (HIV) infection. Reproductive and sexual factors include multiple sexual partners and usage of oral contraceptive (OC) pills. In order to prevent cervical cancer, it is suggested to administer HPV Vaccine as The International Federation of Gynecology and Obstetrics (FIGO) recommendation [4], [5]. Patient had no multiple sexual partners. Therefore, we conclude that early sexual intercourse done by patient was the evident risk factor.

Cervical cancer ranged from stage I to IV. We suggested stage IB2 because we assessed the tumor size was 2 cm until 4 cm. Additionally, we did not find any spreading to nearby lymph nodes nor distant sites [5].

The treatment for stage IB was divided into radical trachelectomy or radical hysterectomy [5]. Radical trachelectomy was preferred for the woman who wanted to preserve fertility. Radical hysterectomy removed the uterus, cervix, the upper part of the vaginal and surrounding ligaments supporting the uterus such as round, broad and uterosacral ligaments. Radiation was an adjunct therapy for cervical cancer. In this case, we conducted a radical hysterectomy and the transposition of the ovary. Additionally, the transposition was important to enhance the patient's quality life. During the hysterectomy procedure, we also did exploration in the abdominal cavity to make sure there were no nodules in other organs.

**Fig. 3:**
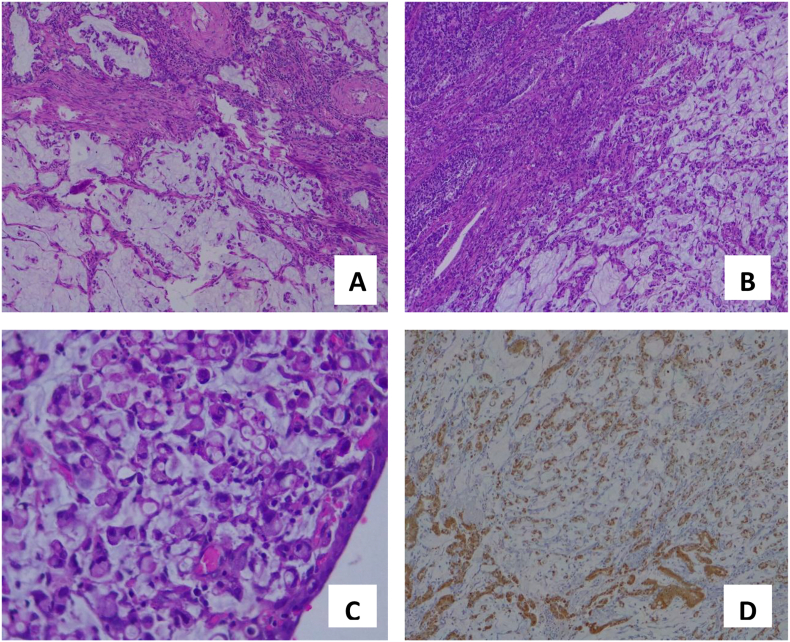
A-C. Histopathology examination. More than 50% tumor cells are arranged in loose clusters and individual cells with intracytoplasmic mucin and perpherally located nuclei, imparting a signet-ring appearance. D. IHC p16 block type positive.

Cervical adenocarcinoma are classified into HPV-associated (usual and mucinous type) and HPV-independent (gastric, clear cell, and mesonephric type). The SRCC was a subtype of mucinous adenocarcinoma and it was commonly originated from gastrointestinal tract malignancy. However, PSRCC of the uterine cervix is a rare case. This tumor is and HPV-associated cervical adenocarcinoma, as it supports by a block type positive p16 IHC staining (Fig. 3D). This rare histopathology feature was reported firstly in 1990 and currently, less than 30 cases reported worldwide [6]. There were no specific clinical symptoms regarding this type, as already known post-coital bleeding is the most complaint of cervical cancer. The histopathology and intraoperative findings emphasized that SRCC is primary from cervical.

**Fig. 4:**
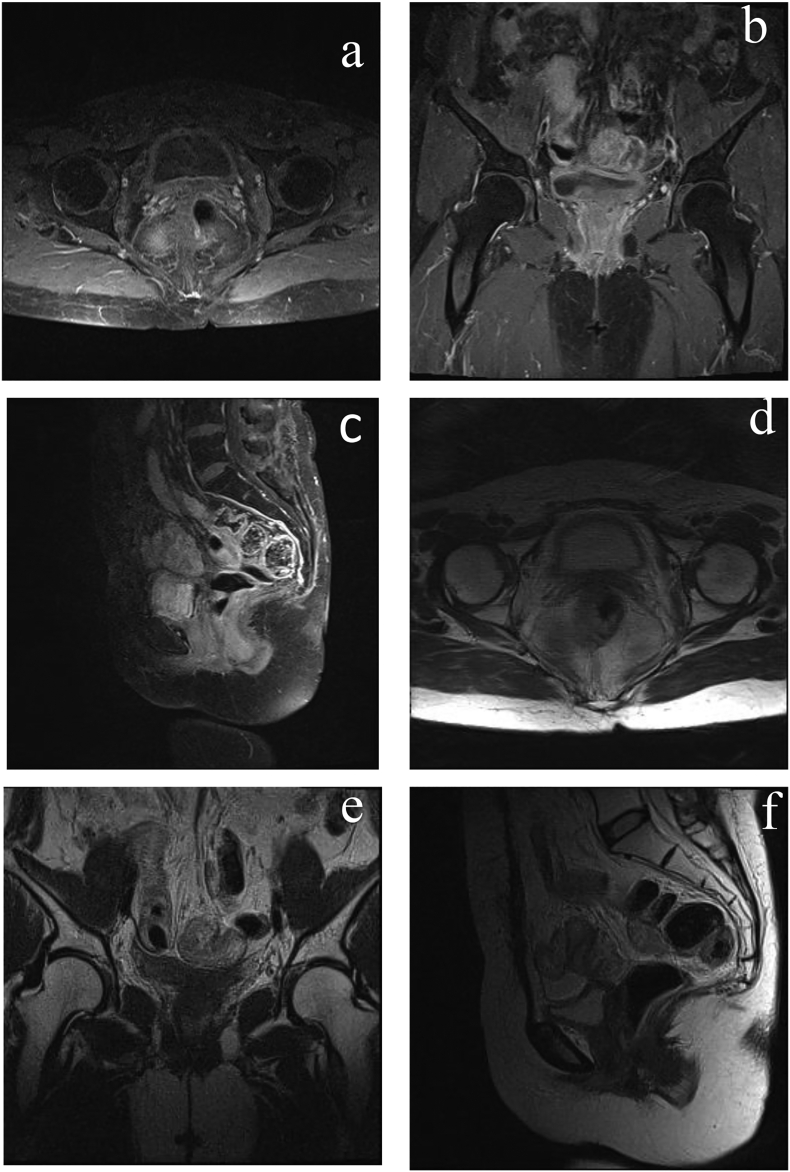
(a to f). Abdominal magnetic resonance imaging with contrast taken after radical hysterectomy and series radiotherapy was completed. Seven months followed-up.

Imaging studies were important to evaluate the therapy. After the radiation was completed, Magnetic Resonance Imaging (MRI) with contrast found no involvement of the other organs. This suggested the effectiveness of the therapy that combined radical hysterectomy and radiation. Hasnawi et al. also reported in their case study PSRCC with metastasis in one of the pelvic lymph nodes were administered radiotherapy alone, both external and internal [7].

The treatment of PSRCC is challenging [8]. There is no standardized recommendation. But in the early stage of the disease, surgical procedures followed by either radiotherapy or combined chemotherapy could be an option [1]. The prognosis of SRCC tends to be poor. Compared to previous reports, the outcome of PSRCC is dominantly related to the cancer staging. No evidence of disease (NED) is reported from six months until more than ten years in the early stage. While in the advanced stage, the NED is reported until six months in one case, and died of the disease varies between seven weeks until nineteen months [9]. In our case, we still need further monitoring, it would be significantly different between treatment and prognosis due to PSRCC of the cervix and metastatic, so the metastasis should be excluded [10].

## 4 Conclusion

PSRCC of the uterine cervix was established from intraoperative findings and pathological examination. The radical hysterectomy adjunct to radiation was effective in the treatment of the PSRCC of the uterine cervix.

## Patient's perspective

I had complaints of bleeding after intercourse for the past year. The doctor advised me to do an IVA examination and was given TCA therapy. During the follow up, the doctor informed me that there was a feature of malignancy in the cervix. My husband and I discussed and decided on surgery and radiation therapy as doctor's recommendation. I followed all the procedures and so far, my condition is good.

## Provenance and peer review

Not commissioned, externally peer-reviewed.

## Sources of funding

This research did not receive any specific grant from funding agencies in the public, commercial, or not-for-profit sectors.

## Ethical approval

This study was reviewed and approved by the Institutional Review Board and Ethical Committee Dr. Cipto Mangunkusumo, a national reference, and teaching hospital. Patient medical records were maintained under applicable medical ethical standards.

## Patient consent

Written informed consent was obtained from the patient for publication of this case report and accompanying images. A copy of the written consent is available for review by the Editor-in-Chief of this journal on request.

## Author contribution

Gatot Purwoto: conceptulization, methodology, resources, supervision.

Gatot Purwoto, Kartiwa Hadi Nuryanto, Kelli Julianti: writing-original draft preparation, investigation.

Taufik Agung Wibowo, Tantri Hellyanti, Danny Maesadatu Syaharutsa, Kelli Julianti: visualization, writing-review and editing.

Gatot Purwoto, Taufik Agung Wibowo, Tantri Hellyanti: supervision, data curation, editing.

## Research registration

None declared.

## Guarantor

Gatot Purwoto.

## Declaration of competing interest

The authors declare that we have no financial or personal relationship that may have inappropriately influenced us in writing this article.
